# LigaSure Impact™ versus conventional dissection technique in pylorus-preserving pancreatoduodenectomy in clinical suspicion of cancerous tumours on the head of the pancreas: study protocol for a randomised controlled trial

**DOI:** 10.1186/1745-6215-12-162

**Published:** 2011-06-29

**Authors:** Tobias Gehrig, Phillip Knebel, Verena Scheel, Ulf Hinz, Christoph M Seiler, Beat P Müller-Stich, Markus W Büchler, Carsten N Gutt

**Affiliations:** 1 Department of General, Abdominal and Transplantation Surgery, University of Heidelberg, Im Neuenheimer Feld 110, 69120, Heidelberg, Germany; 2 Department of General, Visceral, Thoracic and Vascular Surgery, Memmingen Hospital, Memmingen, Germany

## Abstract

**Background:**

The pp-Whipple procedure requires extensive preparation. The conventional preparation technique is done with scissors for dissection and ligatures, and with clips and sutures for hemostasis. This procedure is very time-consuming and requires numerous changes of instruments. The LigaSure™ device allows dissection and hemostasis for preparation with one instrument. Up to now there has been no comparison of the two techniques with regard to operating time and the patients' outcome. It is still unclear which technique has the optimal benefit/risk ratio for the patient.

**Methods/Design:**

A single-center, randomized, single-blinded, controlled superiority trial to compare two different techniques for dissection in a pp-Whipple procedure. 102 patients will be included and randomized pre-operatively. All patients aged 18 years or older scheduled for primary elective pp-Whipple procedure who signed the informed consent will be included. The primary endpoint is the operating time of the randomized technique. Control Intervention: Conventional dissection technique; experimental intervention: LigaSureTM dissection technique. Duration of study: Approximately 15 months; follow up time: 3 years. The trial is registered at German ClinicalTrials Register (DRKS00000166).

## Background

The procedure was originally described by Alessandro Codivilla in 1898, A.O. Whipple improved it in 1935. The Whipple procedure is the standard method for therapy of cancerous tumours, inflammation and stenosis near the head of the pancreas. In the classic Whipple-procedure (c-Whipple) the head of the pancreas, the duodenum, the regional lymph nodes, the gastric antrum, the gallbladder, and the distal bile duct are removed. The pylorus-preserving-Whipple procedure (pp-Whipple) was established by Traverso and Longmire in 1978. During this procedure the gastric antrum is not removed. In recent years the pp-Whipple procedure is preferred because several studies have shown that the classic Whipple procedure is not superior to the pp-Whipple procedure regarding the oncological outcome or peri- and postoperative complication rates [1-5].

About 300 patients are operated on following the pp-Whipple procedure at the department each year. As the pancreas is fed by many vessels [6], it is necessary to use lots of ligatures, clips and sutures for hemostasis after dissection. This dissection technique is very time-consuming and requires numerous changes of instruments. The use of high-frequency feedback-controlled electrothermal bipolar vessel sealant technology, known as the LigaSure™ Vessel Sealing System (LVSS), is a new alternative for dissection and hemostasis. The bipolar vessel dissection devices require no change of instruments for dissection and hemostasis [7-10]. The current is provided by a special HF-generator and contains a very high capacity with a low voltage. The body's proteins, such as collagen and elastin, are converted so a permanently sealed zone results. As only the tissue between the branches is sealed, lateral thermic tissue damages can be limited to a minimum. Several authors describe a tendency of reduced intraoperative blood loss and transfused blood preservations [11-13]. Other trials show reduced operating time using the LVSS in several surgical procedures, such as thyroid, gynecology, urology and haemorrhoidectomy surgery [9,11,13,14].

Correct dissection in the operation field is very important to avert secondary bleeding or other complications, which might cause re-operation or elevate the patients' morbidity and mortality [15].

## Methods/Design

### Aim of study

The comparison of LVSS versus conventional dissection technique in pylorus-preserving pancreatoduodenectomy regarding operation time and complication rates.

### Number of patients needed

**Figure 1:**
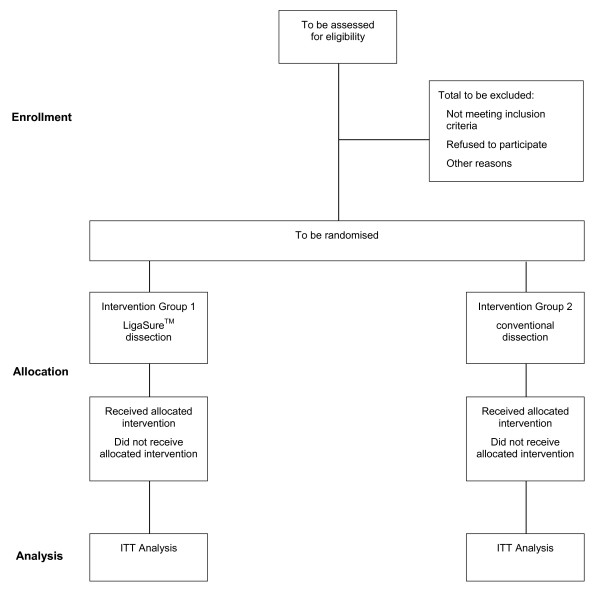
**Flowchart according to CONSORT**.

The sample size calculation is based on a two sided t-test for differences with respect to the primary endpoint. The data evaluation and comparison of seven patients operated on with the LVSS versus seven patients operated on with the conventional dissection technique showed a reduction of operating time of 40 minutes in the LVSS group. An evaluation of all pp-Whipple operations in 2010 (138 operations) showed a mean operating time of 300 min with a standard deviation of 66 minutes. If the difference in operating time is 40 minutes, there will be an 80% (1-β) chance that a trial involving 88 patients (44 per group) could detect a significant difference at an alpha level of 5%s (SAS 9.1 proc power). To compensate possible drop outs 14 patients (15%) more will be randomized. Therefore 102 patients (51 per group) will be included (Figure 1).

A minimum time saving of 40 minutes would be needed to change the manner of surgical preparation towards the use of the LigaSure™ because of the high costs of this device. A basic configuration for the use of the LigaSure™ device costs nearly € 30.000, additionally € 260 per patient are the costs for the single use device itself. The pilot trial with 14 patients by Gehrig et al. showed a cost reduction of nearly € 800 per patient for a operative time saving of 40 minutes (€ 500 for 30 minutes). Therefore we believe a time saving of at least 40 min would be needed to convince surgeons and hospital managers to consider the purchase and use of this equipment on a rugulary basis.

### Eligibility

#### Inclusion criteria

• Clinical suspicion of a cancerous tumour on the head of the pancreas, relied on imaging and laboratory values

• Aged 18 years or older

• Patients scheduled for primary pp-Whipple procedure

• Informed consent

#### Exclusion criteria

• Participation in another trial with interference of outcome of this study

• Lack of compliance (assessed by the trial investigator)

• Impaired mental state or language problems (patient is not able to read German)

#### Subject withdrawal criteria

• At their own request or at request of the legal representative

• If, in the investigator's opinion (surgeon who performs the dissection), continuation of the trial would be detrimental to the subject's well-being (e.g. bleeding or other independent acute health problems)

All withdrawn patients will be reported in the final results to guarantee maximum transparency.

### Consent

The DISSECT- Trial will be conducted in accordance with the protocol and in compliance with the moral, ethical, and scientific principles governing clinical research as set out in the Declaration of Helsinki 1989 [16] and Good Clinical Practice (http://www.ema.europa.eu/docs/en_GB/document_library/Scientific_guideline/2009/09/WC500002874.pdf). The protocol was approved by the Ethics Committee of the University of Heidelberg (S-061/2009). All Patients who are assigned the pp-Whipple procedure at the Department of General, Abdominal and Transplant Surgery, University of Heidelberg, will be screened for eligibility and informed about the DISSECT trial during a visit prior to treatment. The study procedure, risks, benefits and data management will be clarified in detail before the patients are asked to give their informed consent. After inclusion of the patient in the study, his personal data (height (cm), weight (kg), gender, Karnowsky-Index (0 - 100%), medication of immunsuppression, antibiotics (yes/no), chemotherapy (yes/no)) will be recorded into the CRF. (Table 1)

**Table 1:** Study Visit Schedule

			Follow-up	Follow-up
			
	Day of screening	Day of operation	Visit 1 (day 30post OP ) by phone	Visit 2-8 (European Pancreas Center)
Past medical history*	X			

Informed consent	X			

Personal data**	X			

**Examination of primary endpoints**:				
Operating time		X		

**Examination of primary endpoints**:				
Mortality		X	X	X
Peri- and postoperative complications		X	X	X
Re-intervention			X	X
Intraoperative blood loss		X		
Hospital stay			X	
Reuptake			X	X
Time of anesthesia pre- and postoperative		X		
Intraoperative material consumption		X		
Local recurrence				X
Quality of life	X			X

Safety criteria AE, SAE (2.6)		X	X	X

* study-relevant past medical history, past surgical history

** height (cm), weight (kg), gender, Karnowsky-Index, medication of immunsuppresion, antibiotics, chemotherapy

### Randomization and procedures for minimizing bias

#### Minimizing systemic bias

To achieve comparable groups for known and unknown risk factors randomization will be performed as unstratified block randomization with random block sizes in a 1:1 allocation ratio. Allocation to treatment group will be carried out by the randomization software RITA^® ^[17]. Randomization numbers that become vacant by withdrawal will be reused.

102 patients will be recruited according to the sample size calculation. Randomization will be performed independently by a study nurse of the Clinical Study Center Surgery (KSC) to the conventional group or to the LVSS group. Randomization will be carried out after patient has signed the informed consent and will be documented in the case report file of every patient. Intervention will be scheduled 1-3 days after inclusion depending on the earliest operation appointment possible.

#### Minimizing treatment bias

A standardized operation technique will be used in both groups. The same LigaSure™ device will be used in the LVSS group. The physician responsible needs to have experience of at least 100 pp-Whipple procedures and will be trained and updated every three months to guarantee comparable treatment of patients.

#### Minimizing measurement bias

A study nurse will document and monitor the procedure in the operating theatre. The patients are blinded for the used operation technique. Physicians' blinding is not possible due to the different techniques used during the operation.

### Study treatment

The surgical technique of PPW procedure is highly standardized in the surgical department and consists of the following operative steps [12,13]: After mobilization of the hepatic flexure of the colon, the duodenum and head of the pancreas are separated from the retroperitoneal bed (Kocher maneuver). The gallbladder is removed and the bile duct is divided above the cystic duct entry across the common hepatic duct. Next, the portal vein is exposed. After division of the right gastric artery, the gastroduodenal artery and gastroepiploic vessels, the duodenum distal to the pylorus is divided. The pancreas is transected in front of the portal vein using a scalpel and not with the Ligasure Impact™ device. Dissection of the distal duodenum proximal to the ligament of Treitz is then performed. This is followed by the reconstruction phase, with preparation and resection of the proximal 10-15 cm section of the jejunum at a point that will provide sufficient mobility of the jejunum to reach the right upper quadrant after it is brought through the transverse mesocolon for the biliary and pancreatic anastomosis. Pancreatojejunostomy is performed with two layers, end-to-side, after mobilization of the pancreatic stump. The end-to-side hepatojejunostomy is made with a single layer and is placed about 10-15 cm aboral of the pancreatojejunostomy. Finally, an end-to-side duodenojejunostomy with two layers is performed about 50 cm distal to the pancreatojejunostomy in an antecolic position.

#### Experimental group

**Figure 2:**
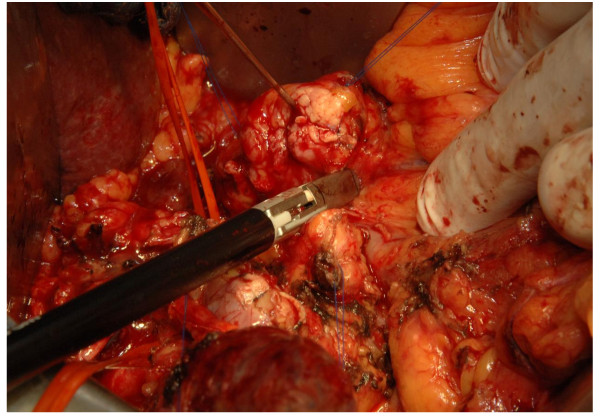
**Dissection with the LVSS in pylorus-preserving pancreatoduodenectomy**.

**Figure 3:**
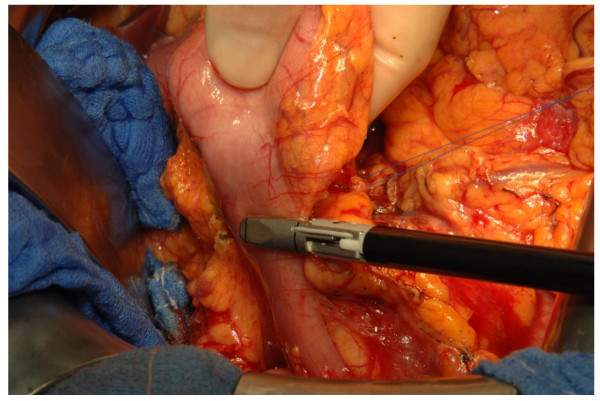
**Transection of the proximal duodenum with the LVSS**.

In the LVSS group, dissection and hemostasis of vessels with a diameter of up to seven millimeters (Figure 2) as well as bowel transection (Figure 3) will be performed with the Ligasure Impact™ device (Valleylab™, Boulder, Colorado, USA). For safety reasons, the transected bowel will be overstitched and larger vessels will be ligated in the LVSS group.

#### Control group

In the conventional group, scissors, ligatures, clips and sutures will be used for dissection and hemostasis. Transection of the small bowel will be done with a Linear Cutter 55 2.0 mm (Ethicon Endo-Surgery, Inc., USA).

### Primary and secondary endpoints

#### Primary endpoint

The primary endpoint will be the operating time of the randomized dissection technique.

Definition of the primary endpoint:

The operation time will be measured from the beginning of the surgical procedure (incision of the skin) to the end of the surgical procedure (closure of the skin). It will be documented in the operation log.

Assessment of the primary endpoint:

The operating time will be assessed postoperatively in the case report file (CRF) by the physician responsible and will be confirmed by an independent study nurse. A comparison to the operation report will ensue.

#### Secondary endpoints

• Perioperative complications:

○ Intraoperative Bleeding

○ Perforation of hollow organs (stomach, small intestine, colon, gallbladder/biliary tract)

○ Lesion in parenchymatous abdominal viscus (liver, spleen, pancreas)

• Postoperative complications:

○ Secondary bleeding/hematoma

○ Wound infection

○ Gastroparesis

○ Postoperative pancreatic fistula (POPF)

○ Intraabdominal abscess

○ Anastomotic leakage of the choledochojejunostomy

○ Anastomotic leakage of the gastrojejunostomy

• Re-intervention (operational/interventional)

• Intraoperative blood loss

• Hospital stay

• Re-hospitalization-rate (30 days postoperative)

• Duration of anesthesia

• Intraoperative material consumption

• Material costs, calculated by the material consumption

• Overall costs of the operation, calculated by the indirect and direct costs during the hospital stay and the costs accumulated 30 days postoperatively

• Local recurrence of disease

• Quality of life analysed in questionnaires (EORTC QLQ-C30, EORTC QLQ PAN26, special questionnaire of the European Pancreas Center, University of Heidelberg)

• Mortality rate

Definitions of secondary endpoints are shown in Table 2.

**Table 2:** Definition of secondary endpoints

Perioperative complications	Complication	Definition
Bleeding	Severe intraoperative bleeding	Mentioned in operation report

Perforation of hollow organs	Perforation of stomach, small intestine, colon or biliary tract/gallbladder	Mentioned in operation report

Lesion in parenchymatous abdominal viscus	Lesion in liver, spleen, pancreas	Mentioned in operation report

Postoperative complications	Complication	Definition

Secondary bleeding/hematoma	postoperative bleeding	Need for more than 2 units of red blood cells within 24 hours after surgery OR Need of surgical treatment (mentioned in re-operation report)
	
	Hemtoma	Radiological (ultrasound, CT) findings positive for reoperation or reopening of the wound

wound infection		Deep and/or superficial according to CDC definition [20]

Gastroparesis	delayed gastric emptying (DGE)	Consensus definition of delayed gastric emptying after pancreatic surgery of the International Study Group of Pancreatic Surgery (ISGPS) [21]

pp-Whipple specific complications	postoperative pancreatic fistula (POPF)	Drain output of any measurable volume of fluid on or after postoperative day 3 with an amylase content greater than 3 times the serum amylase activity. Three different grades of POPF (grades A, B, C) are defined according to the clinical impact on the patient's hospital course [22]
	
	Intraabdominal abscess	Intraabdominal collection of purulent or infected fluids (positiv bacterial culture) requiring radiological (puncture or drainage of purulent fluid) or surgical intervention (re-operation)
	
	anastomotic leakage of choledochojejunostomy	Bilirubin-rich (more than 5000 units) drainage fluid of more than 50 ml per day on or after the 10th postoperative day OR Mentioned in re-operation report
	
	anastomotic leakage of gastrojejunostomy	Radiological findings correlating to gastrojejunostomy insufficiency (e.g. CT with contrast medium withdrawal) OR Mentioned in re-operation report

Further secondary endpoints		Definition

Re-intervention	operational	Reoperation due to any cause

	interventional	Interventional haemostasis or drainages due to any cause

Intraoperative blood loss		Mentioned in operation report

Hospital stay		Time of admission to discharge

Re-hospitalization-rate		Re- hospilization within 30 days after surgery due to any cause

Duration of anesthesia		Mentioned in anesthesia report

Intraoperative material consumption		All materials, which are necessary for surgery

Material costs		Costs, which occurs due to the material consumption during the surgery

Overall costs		Costs include expenditure for personnel and use of operating room as well as for material consumption

Local recurrence of disease		Radiological (ultrasound, CT) findings positive for recurrence of disease

Quality of life		EORTC QLQ-C30, EORTC QLQ PAN26 and special questionnaire of the European Pancreas Center Heidelberg on day 30 (±10days), 90 (±10 days), 180 (±10 days), 1 year (± 1 month), 1,5 years (± 1 month), 2 years (± 1 month), 2,5 years (± 1 month) and 3 years (± 1 month)

Mortality rate		Death to any course until year 3 after operation

Assessment:

1. Perioperative complications of the pp-Whipple procedure will be recorded on the day of operation by an independent study nurse.

2. Postoperative complications of the pp-Whipple procedure will be recorded in patients' discharge data and on visit 1 (30 days after operation) after a standardized telephone interview by a study nurse. A confirmation by the patient's family physician will be requested following any abnormality reported by the patient. The following standard aftercare takes place initially twice quarterly (visit 2, 3 months after operation; visit 3, 6 months after operation) then half-yearly for two years (visit 4, 12 months after operation; visit 5, 18 months after operation; visit 6, 24 months after operation; visit 7, 30 months after operation and visit 8, 36 months after operation) with a clinical examination, imaging (ultrasound, CT, MRT), general and standardized questionnaires (EORTC-QLQ-C30, EORTC-QLQ-PAN26 and a special questionnaire from the European Pancreas Center) and laboratory value control in the European Pancreas Center at the surgical department.

### Safety aspects

#### Specification of safety variables

##### Training for surgeons

In each operation there will be a surgeon (senior surgeon) who has experience of at least 100 pp-Whipple procedures. The operation will be carried out as a standard procedure; the LigaSure™ device has been used in the department for several years already.

##### Concomitant medication

Concomitant medication will not be recorded because the primary operating time of the two dissection techniques is a local and technical endpoint. Therefore, a systemic pharmacological interaction with the medication of the patient will be very unlikely.

##### Past medical history

Prior and concomitant illness of the patients will be documented in the CRF. The category of the primary disease (reason for pp-Whipple procedure) is one of the variables to be analyzed for baseline comparability.

##### Adverse events and serious adverse events

AEs will be reported to the principal investigator in regular intervals during the course of the study and will be documented in the CRF. It is necessary to note the date, the symptoms, beginning and end, treatment, severity (mild, moderate, severe) and the context of the operation (except those events detected in the endpoints).

SAEs which meet one of the definitions of the secondary endpoints are treated as SAEs regarding their documentation but do not have to be reported to the sponsor (Covidien GmbH, Germany) and principal investigator (Prof. Dr. MW Büchler, Chairman of the Department of General, Visceral and Transplantation Surgery, University Hospital of Heidelberg) within 24 hours. They will be reported to the principal investigator in regular intervals throughout the study. The surgical trial coordinator will also cross-check the SAEs/AEs of all patients.

## Analysis

Comparisons will be made of the primary endpoints of both intervention groups for all randomized patients who underwent surgery involving the pp-Whipple procedure. Patients will be analysed as randomized applying the ITT principle [18]. In addition, a per-protocol analysis will be performed, including patients who are strictly treated according to the study protocol.

The outcome measures of the primary endpoint will be tested confirmatory applying an analysis of covariance with treatment as factor and age and BMI as continuous covariates.

Secondary endpoints will be analysed in a descriptive manner. Graphically methods will be used by means of box- and scatter- plots. For all continuous secondary endpoints a t-test will be applied, possible differences of categorical secondary endpoints will be analysed using chi-square tests. All p-values will be used as descriptive statistics only without any confirmatory value.

The secondary endpoint Quality of Life (EORTC QLQ-C30, EORTC QLQ PAN26) will be anlysed by an application of analysis of covariance that adjusts for age and EORTC QLQ-C30, EORTC QLQ PAN26 before surgery.

The following procedure will be applied for missing data:

If at least half of the items from the scale have been answered, the missing items are replaced by the average of those items that are not missing (calculated average rounded to the nearest integer). If less than half of the items from the scale have been answered, the scale is set to missing [19].

If it is evident that missing items or missing scales are related to a worsening in physical functioning, the missing values are replaced by the worst value measured in the respective intervention group at the respective time point.

The average EORTC QLQ-C30, EORTC QLQ PAN26 score can be calculated, if at least one measurement at day 30 (±10days), 90 (±10 days), 180 (±10 days), 1 year (± 1 month), 1,5 years (± 1 month), 2 years (± 1 month), 2,5 years (± 1 month) or 3 years (± 1 month) after surgery is available; if no measurement of the EORTC QLQ-C30 or EORTC QLQ PAN26 after surgery is available, the secondary endpoint is missing and the patient is therefore not included into the analysis.

All statistical analyses will be performed using SAS^® ^software, Version 9.1 (or higher) of the SAS System for Unix (SAS Institute Inc., Cary, NC, USA).

## Study organization

After approval of the protocol by the local ethics committee of the University of Heidelberg, the trial was internationally registered at Germanctr.de (DRKS00000166). All patients scheduled for a pp-Whipple procedure in the Department of General, Visceral and Transplantation Surgery, University Hospital of Heidelberg, will be referred to and screened by members of the Clinical Study Center Surgery (KSC). The results of the screening will be recorded in the screening-log.

Approximately 300 patients per year undergo a pp-Whipple procedure in the Department of General, Visceral and Transplantation Surgery, University Hospital of Heidelberg. The estimated time frame to randomize 102 patients will be approximately 15 months.

The sponsor of the DISSECT trial is Covidien GmbH (Germany).

The independent data management and statistical analysis will be carried out by the Institute of Medical Biometry and Informatics (IMBI) of the University of Heidelberg according to a prespecified Statistical Analysis Plan. It controls the completeness and correctness of the CRF as well as the administration.

The principal investigator has the right to terminate the trial and to remove all trial material from the trial centre at any time in consultation with the Clinical Study Team Leader and the Biostatistician. Reasons that may require a termination of the trial include the following:

• The incidence or severity of adverse events in the trial indicates a potential health hazard caused by the study treatment

• It appears that the patient's enrolment is unsatisfactory with respect to quality or quantity or data recording is severely inaccurate or incomplete

• External evidence that renders the necessity to terminate the trial

## Abbreviations

IMBI: Institute for Medical Biometry and Informatics; c-Whipple: classic-Whipple; pp-Whipple: pylorus-preserving-Whipple; LVSS: LigaSure™ Vessel Sealing System; HF: high frequency; SAS: Statistical Analysis Systems; CRF: case report file; RITA: Real Time Interactive Applications; KSC: Clinical Study Venter Surgery; PPW: pylorus-preserving Whipple; POPF: postoperative pancreatic fistula; EORTC: European Organisation for Research and Treatment of Cancer; QLQ-C30: Quality of Life Questionnaire-Core 30; QLQ-PAN26: Quality of Life Questionnaire- Pancreatic Cancer Module 26; CT: computed tomography; MRT: magnetic resonance tomography; AEs: adverse events; SAEs: serious adverse events; ITT: intention to treat; CDC: Centers for Disease Control and Prevention; DGE: delayed gastric emptying; ISGPS: International Study group of Pancreatic Surgery.

## Competing interests

The principal investigator received a grant from Covidien GmbH (Germany). There are no restrictions on the publications.

## Authors' contributions

TG, BPM, CS and CNG are responsible for the study design, definitions of the primary and secondary endpoints, preparation of the protocol and manuscript. UH and CS are responsible for the sample size calculation. TG, PK and VS carried out the literature research and are responsible for the preparation of the manuscript. All authors read and approved the final manuscript.
